# Ribonucleic acid-binding protein CPSF6 promotes glycolysis and suppresses apoptosis in hepatocellular carcinoma cells by inhibiting the BTG2 expression

**DOI:** 10.1186/s12938-021-00903-6

**Published:** 2021-07-03

**Authors:** Yang Liu, Hongbo Zou, Qichao Xie, Lan Zou, Rui Kong, Bijing Mao

**Affiliations:** 1 Department of Critical Medicine, The Fifth Affiliated Hospital of Sun Yat-Sen University, Zhuhai, 519000 China; 2 Department of Oncology, The Third Affiliated Hospital of Chongqing Medical University, No.1, Shuanghu branch Road, Yubei District, Chongqing, 401120 China

**Keywords:** CPSF6, BTG2, Glycolysis, Hepatocellular carcinoma cells, AKT/ERK/NFκB pathway

## Abstract

Hepatocellular carcinoma (HCC) is currently the sixth most common malignancy and the second major cause of tumor-related deaths in the world. This study aimed to investigate the role of cleavage and polyadenylation factor-6 (CPSF6) and B-cell translocation gene 2 (BTG2) in regulating the glycolysis and apoptosis in HCC cells. The RNA and protein expression of CPSF6 and BTG2 in normal hepatocyte and HCC were, respectively, detected by reverse transcription quantitative real-time polymerase chain reaction (RT-qPCR) analysis and Western blot analysis. The viability and apoptosis of transfected Huh-7 cells were, respectively, analyzed by 3-(4,5-dimethylthiazol-2-yl)-2,5-diphenyltetrazolium bromide (MTT) assay and terminal deoxynucleotidyl transferase (TdT) dUTP Nick-End Labeling (TUNEL) assay. The expression of apoptosis-related proteins and HK-2 in transfected Huh-7 cells was also detected by Western blot analysis. The levels of glucose and lactate in the culture supernatant of transfected Huh-7 cells were, respectively, detected with the glucose assay kit and lactate assay kit. The interaction of CPSF6 and BTG2 was confirmed by RNA binding protein immunoprecipitation (RIP) assay. As a result, CPSF6 expression was increased while BTG2 expression was decreased in Huh-7 cells. Interference with CPSF6 suppressed the viability and glycolysis, and promoted the apoptosis of Huh-7 cells. Furthermore, CPSF6 interacted with BTG2 and interference with CPSF6 upregulated the BTG2 expression and inhibited the protein kinase B (AKT)/extracellular signal-regulated kinase (ERK)/nuclear factor (NF)-κB pathway. Interference with BTG2 could partially reverse the above cell changes caused by interference with CPSF6. In conclusion, CPSF6 inhibited the BTG2 expression to promote glycolysis and suppress apoptosis in HCC cells by activating AKT/ERK/NF-κB pathway.

**Supplementary Information:**

The online version contains supplementary material available at 10.1186/s12938-021-00903-6.

## Introduction

Hepatocellular carcinoma (HCC) was a pathological type with the highest incidence in liver malignant tumor, accounting for about 85% of primary liver cancer. The incidence of HCC was the sixth highest among all malignancies and the second highest among all tumor-associated causes of death [1, 2]. Despite the improvement and optimization of HCC treatment protocols in recent years, the poor prognosis of HCC patients remains unchanged. The high recurrence rate after treatment and the invasion and metastasis inside and outside the liver are the main reasons for the failure of HCC therapy [3–5]. Therefore, it is necessary to screen new and effective diagnostic and therapeutic targets for HCC to open up new approaches for early diagnosis and treatment of HCC.

Cleavage and polyadenylation factor-6 (CPSF6), as one of the cleavage factor Im (CFIm) subunits during alternative polyadenylation (APA) of mRNA, has been thought to be related to the occurrence and development of cancer in recent years [6]. HER2-overexpressing and triple-negative subtypes depend on CPSF6 for viability and tumorigenic capacity in aggressive breast cancer cells of luminal B, and CPSF6 is overexpressed in human breast cancer cases, which is associated with poor prognosis [7]. In breast cancer, downregulation of CPSF6 leaded to the decrease of proliferation, migration and invasion of cells [8]. In the colon cancer cells expressing Snail1, expression of CPSF6 and splicing factor proline/glutamine-rich (SFPQ) is higher than that of the control group, suggesting the pro-oncogenic effect of CPSF6 [9]. ENCORI (http://starbase.sysu.edu.cn/index.php) shows that CPSF6 is upregulated in patients with HCC and is associated with poor prognosis (Additional file 1: Figure S1A, B).

According to the prediction of ENCORI, CPSF6 as an RNA binding protein can bind and regulate B-cell translocation gene 2 (BTG2). BTG2, a member of the BTG/TOB family, was the first gene to be identified as a recognized tumor suppressor gene with anti-proliferation properties. BTG2 was identified as a direct and functional target of miR-6875-3p. miR-6875-3p promoted the epithelial–mesenchymal transition (EMT) and promotes proliferation and metastasis of HCC cells by down-regulating BTG2 expression [10]. Moreover, upregulated expression of BTG2 inhibited the migration, invasion, EMT and glycolysis in lung adenocarcinoma (LUAC) cells and tumor growth [11]. In addition, BTG2 expression in liver cancer cells was obviously declined and upregulation of BTG2 expression suppressed the proliferation and invasion while enhanced the apoptosis of HepG2 cells [12]. Kaplan–Meier curves indicate that liver cancer patients with high BTG2 expression have significantly longer survival time than those with low BTG2 expression (Additional file 2: Figure S2).

Therefore, this study was to explore the role of CPSF6 and BTG2 in regulating the glycolysis and apoptosis in HCC cells and the underlying mechanism.

## Results

### Expression level of CPSF6 in HCC cells

**Fig. 1:**
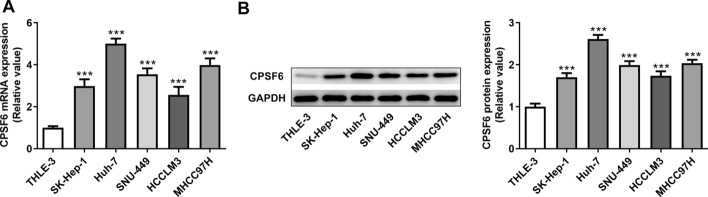
Expression level of CPSF6 in hepatocellular carcinoma cells. **A** CPSF6 mRNA expression in hepatocellular carcinoma cells was detected by RT-qPCR analysis. **B** CPSF6 protein expression in hepatocellular carcinoma cells was determined by Western blot analysis. ***P < 0.001 vs. THLE-3 group. *N* = 3

The mRNA expression of CPSF6 was obviously upregulated in hepatocellular carcinoma cells compared with THLE-3 cells and the mRNA expression of CPSF6 in Huh-7 cells was the highest (Fig. 1A). The protein expression of CPSF6 in those cells was consistent with mRNA expression (Fig. 1B). Therefore, Huh-7 cell line was selected.

### Interference with CPSF6 promotes apoptosis of HCC cells

**Fig. 2:**
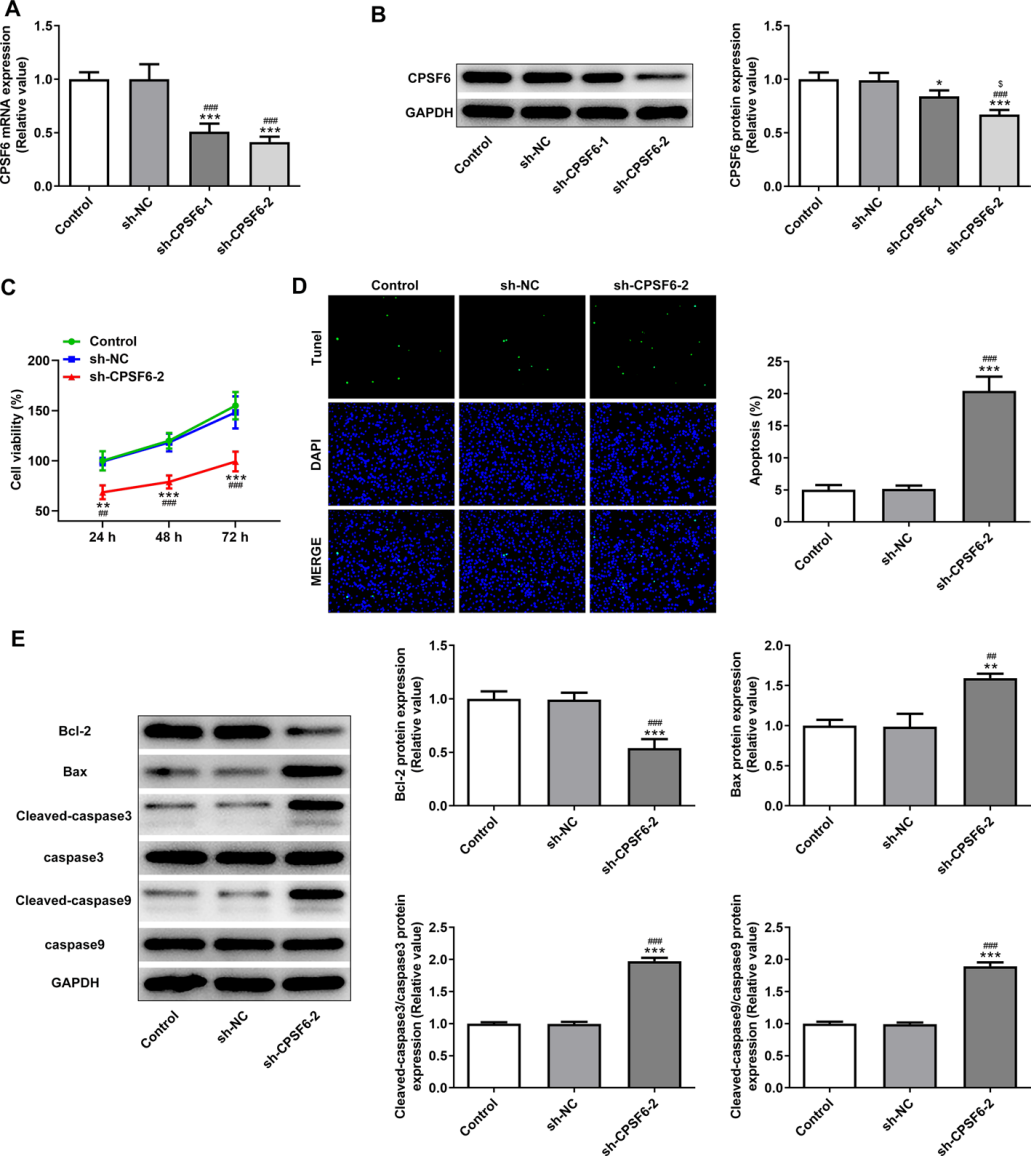
Interference with CPSF6 promotes apoptosis of hepatocellular carcinoma cells. **A** CPSF6 mRNA expression in Huh-7 cells transfected with sh-CPSF6-1/2 was detected by RT-qPCR analysis. **B** CPSF6 protein expression in Huh-7 cells transfected with sh-CPSF6-1/2 was determined by Western blot analysis. *P < 0.05 and ***P < 0.001 vs. Control group. ^###^*P* < 0.001 vs. sh-NC group. ^$^*P* < 0.05 vs. sh-CPSF6-1 group. **C** The viability of Huh-7 cells transfected with sh-CPSF6-2 was analyzed by MTT assay. **D** The apoptosis of Huh-7 cells transfected with sh-CPSF6-2 was detected by TUNEL assay. **E** The expression of apoptosis-related proteins in Huh-7 cells transfected with sh-CPSF6-2 was analyzed by Western blot analysis. **P < 0.01 and ***P < 0.001 vs. Control group. ^##^*P* < 0.01 and ^###^*P* < 0.001 vs. sh-NC group. *N* = 3

After Huh-7 cells were transfected with sh-CPSF6-1/2, CPSF6 mRNA expression was markedly decreased and lower in sh-CPSF6-2 group (Fig. 2A), which was consistent with the results of western blot analysis (Fig. 2B), thereby sh-CPSF6-2 was selected for subsequent experiment. Interference with CPSF6 significantly suppressed the viability (Fig. 2C) and obviously promoted the apoptosis (Fig. 2D) of Huh-7 cells. Interference with CPSF6 suppressed the Bcl-2 expression while enhanced the expression of Bax, cleaved-caspase3 and cleaved-caspase9 (Fig. 2E).

### Interference with CPSF6 inhibits glycolysis of HCC cells

**Fig. 3:**
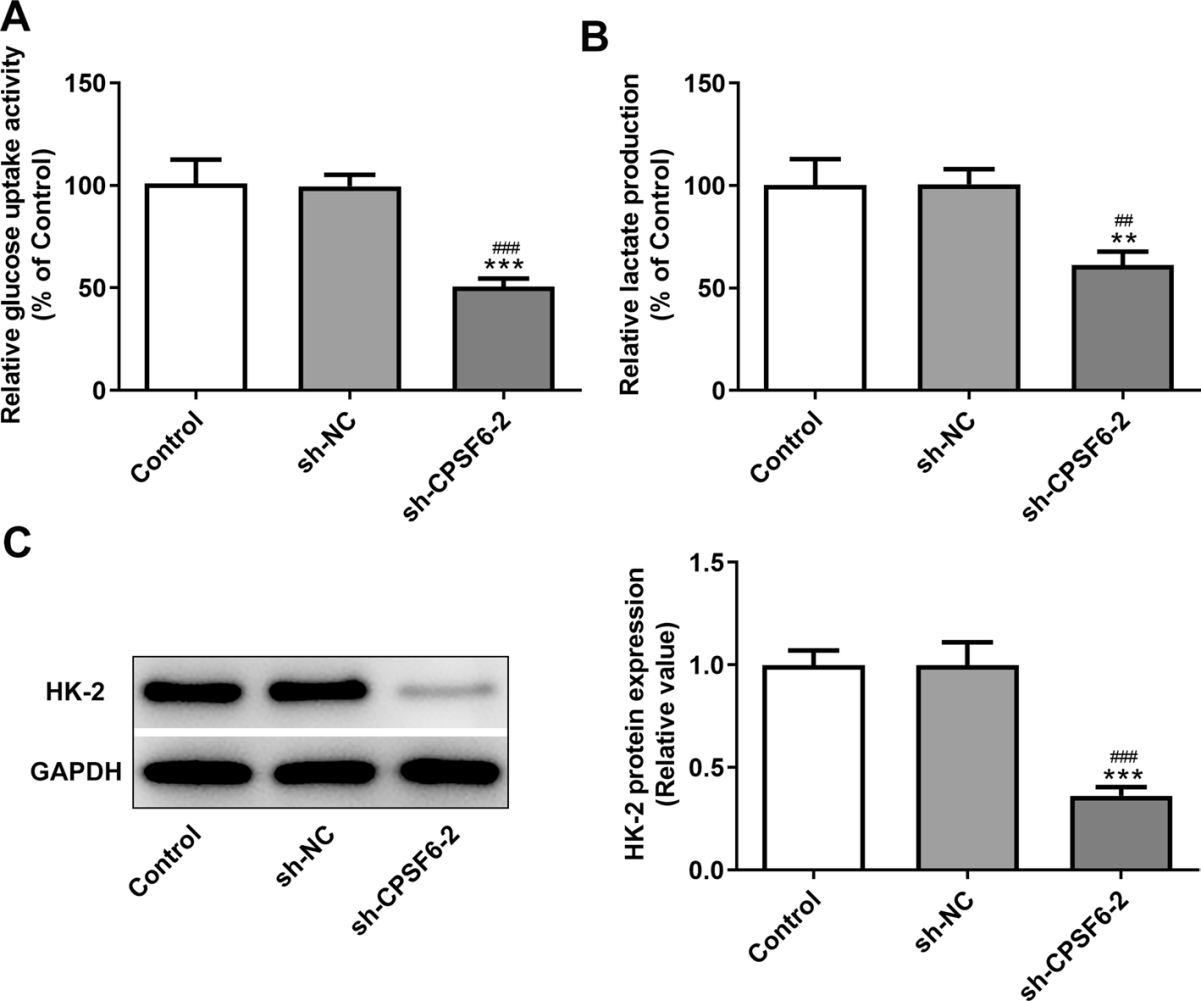
Interference with CPSF6 inhibits glycolysis of hepatocellular carcinoma cells. **A** The glucose uptake activity of Huh-7 cells transfected with sh-CPSF6-2 was analyzed by glucose assay kit. **B** The lactate production of Huh-7 cells transfected with sh-CPSF6-2 was analyzed by lactate assay kit. **C** HK-2 expression in Huh-7 cells transfected with sh-CPSF6-2 was detected by Western blot analysis. **P < 0.01 and ***P < 0.001 vs. Control group. ^##^*P* < 0.01 and ^###^*P* < 0.001 vs. sh-NC group. *N* = 3

After Huh-7 cells were transfected with sh-CPSF6-2, the ability of glucose uptake (Fig. 3A) and lactate production (Fig. 3B) was obviously declined. The level of glycolytic enzyme HK-2 was also lowly expressed in sh-CPSF6-2 transfected Huh-7 cells (Fig. 3C).

### CPSF6 as an RNA binding protein can bind and regulate BTG2 expression

**Fig. 4:**
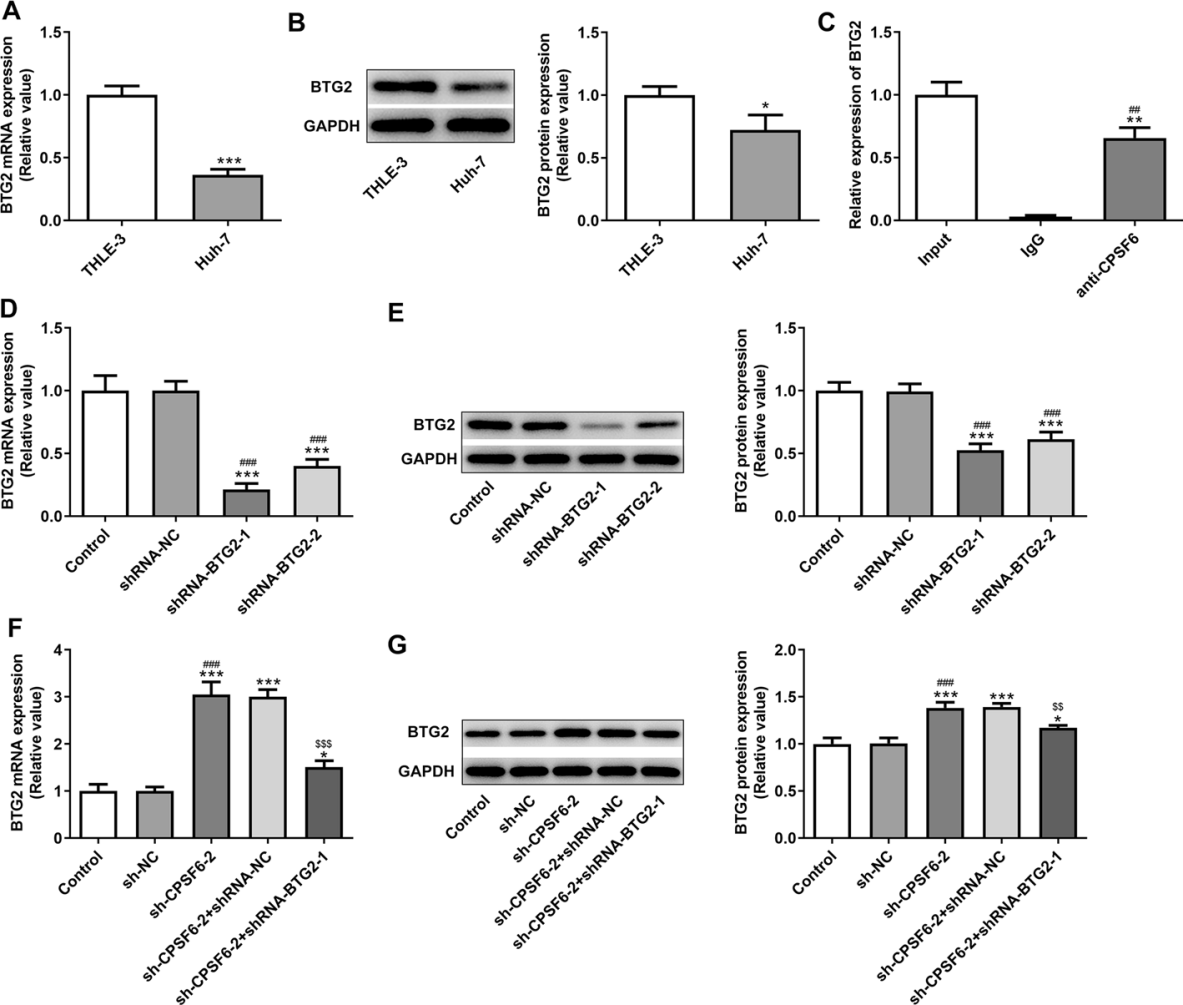
CPSF6 as an RNA binding protein can bind and regulate BTG2 expression. **A** BTG2 mRNA expression in Huh-7 cells was detected by RT-qPCR analysis. **B** BTG2 protein expression in Huh-7 cells was determined by Western blot analysis. *P < 0.05 and ***P < 0.001 vs. THLE-3 group. **C** RIP assay confirmed that CPSF6 was interacted with BTG2. **P < 0.01 vs. Input group. ^##^*P* < 0.01 vs. IgG group. **D** BTG2 mRNA expression in Huh-7 cells transfected with shRNA-BTG2-1/2 was detected by RT-qPCR analysis. **E** BTG2 protein expression in Huh-7 cells transfected with shRNA-BTG2-1/2 was determined by Western blot analysis. ***P < 0.001 vs. Control group. ^###^*P* < 0.001 vs. shRNA-NC group. **F** BTG2 mRNA expression in Huh-7 cells transfected with sh-CPSF6-2 and shRNA-BTG2-1 was detected by RT-qPCR analysis. **G** BTG2 mRNA expression in Huh-7 cells transfected with sh-CPSF6-2 and shRNA-BTG2-1 was detected by Western blot analysis. *P < 0.05 and ***P < 0.001 vs. Control group. ^###^*P* < 0.001 vs. sh-NC group. ^$$^*P* < 0.01 and ^$$$^*P* < 0.001 vs. sh-CPSF6-2 group. *N* = 3

The mRNA expression of BTG2 was remarkably declined in Huh-7 cells compared with THLE-3 cells (Fig. 4A), which was consistent with the protein expression of BTG2 (Fig. 4B). RIP assay indicated that CPSF6 was interacted with BTG2 (Fig. 4C). The mRNA expression of BTG2 was obviously reduced in Huh-7 cells transfected with shRNA-BTG2-1/2 (Fig. 4D), which was also observed in protein expression of BTG2 (Fig. 4E), thereby shRNA-BTG2-1 was chosen. Interference with CPSF6 significantly upregulated the mRNA and protein expression of BTG2, which was partially reversed by interference with BTG2 (Fig. 4F, G).

### Interference with BTG2 can partially reverse the effects of interference with CPSF6 on apoptosis and glycolysis of HCC cells

**Fig. 5:**
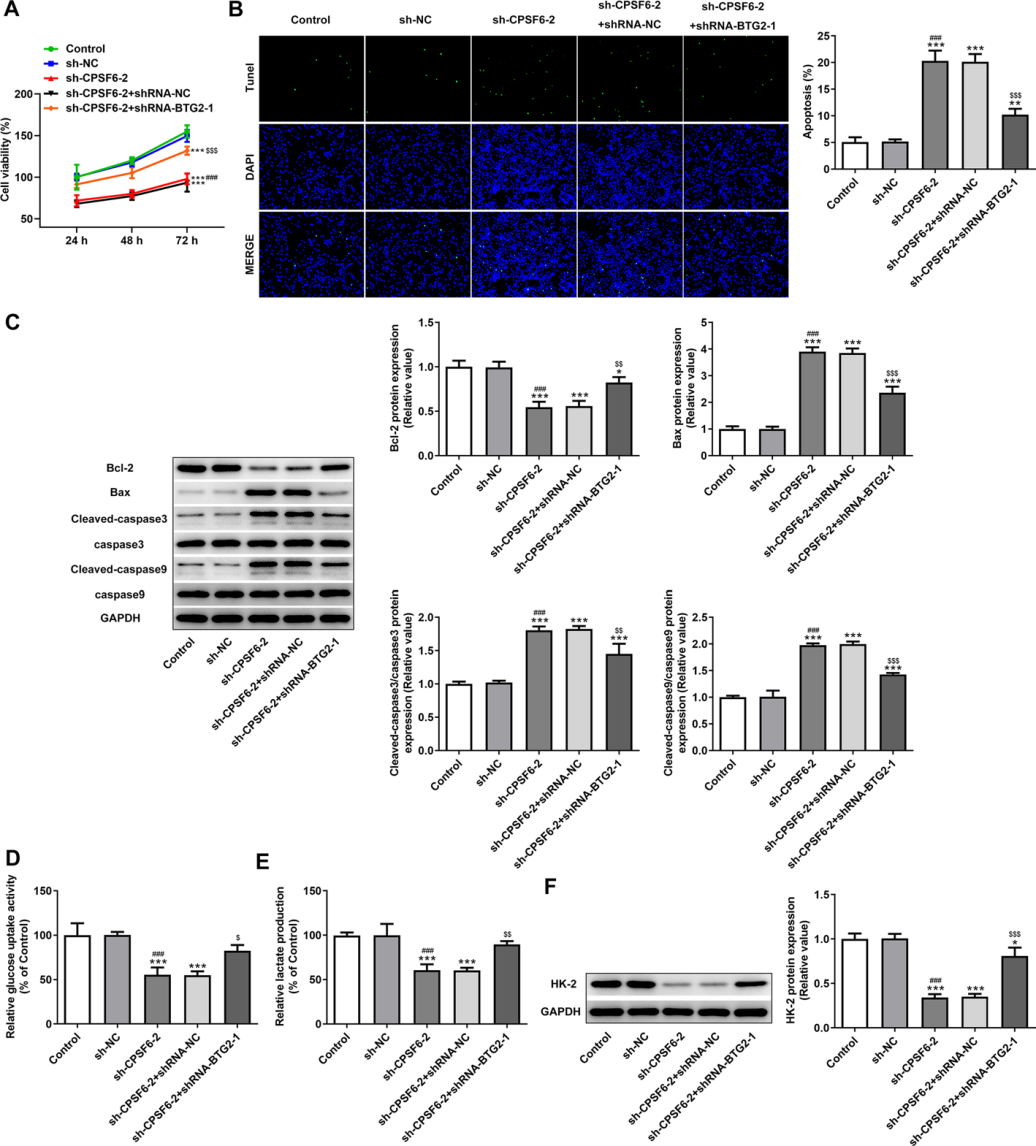
Interference with BTG2 can partially reverse the effects of interference with CPSF6 on apoptosis and glycolysis of hepatocellular carcinoma cells. **A** The viability of Huh-7 cells transfected with sh-CPSF6-2 and shRNA-BTG2-1 was detected by MTT assay. **B** The apoptosis of Huh-7 cells transfected with sh-CPSF6-2 and shRNA-BTG2-1 was analyzed by TUNEL assay. **C** The expression of apoptosis-related proteins in Huh-7 cells transfected with sh-CPSF6-2 and shRNA-BTG2-1 was determined by Western blot analysis. **D** The glucose uptake activity of Huh-7 cells transfected with sh-CPSF6-2 and shRNA-BTG2-1 was analyzed by glucose assay kit. **E** The lactate production of Huh-7 cells transfected with sh-CPSF6-2 and shRNA-BTG2-1 was analyzed by lactate assay kit. **F** HK-2 expression in Huh-7 cells transfected with sh-CPSF6-2 and shRNA-BTG2-1 was determined by Western blot analysis. *P < 0.05, **P < 0.01 and ***P < 0.001 vs. Control group. ^###^*P* < 0.001 vs. sh-NC group. ^$^*P* < 0.05, ^$$^*P* < 0.01 and ^$$$^*P* < 0.001 vs. sh-CPSF6-2 group. *N* = 3

Interference with BTG2 markedly promoted the viability (Fig. 5A) and suppressed the apoptosis (Fig. 5B, C) of Huh-7 cells transfected with sh-CPSF6-2. Interference with BTG2 significantly increased the Bcl-2 expression and obviously decreased the expression of Bax, cleaved-caspase3 and cleaved-caspase9 in Huh-7 cells transfected with sh-CPSF6-2 (Fig. 5D). Interference with BTG2 notably improved the ability of glucose uptake (Fig. 5E) and lactate production (Fig. 5E), and also markedly increased the glycolytic enzyme HK-2 (Fig. 5F) in Huh-7 cells transfected with sh-CPSF6-2.

### Interference with CPSF6 inhibits AKT/ERK/NF-κB signaling by upregulating BTG2 expression

**Fig. 6:**
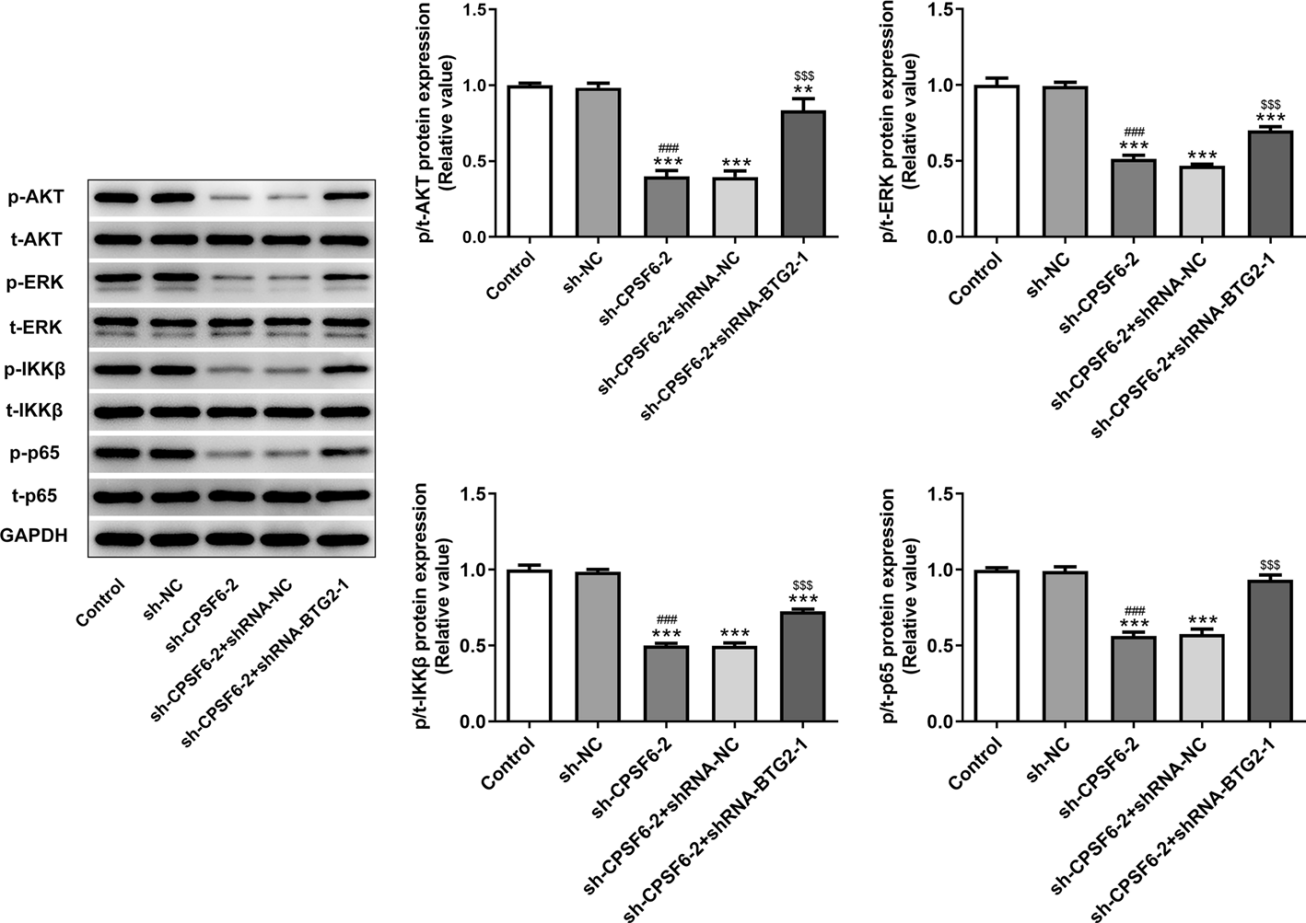
Interference with CPSF6 inhibits AKT/ERK/NF-κB signaling by upregulating BTG2 expression. The expression of AKT/ERK/NF-κB signaling in Huh-7 cells transfected with sh-CPSF6-2 and shRNA-BTG2-1 was determined by Western blot analysis. **P < 0.01 and ***P < 0.001 vs. Control group. ^###^*P* < 0.001 vs. sh-NC group. ^$$$^*P* < 0.001 vs. sh-CPSF6-2 group. *N* = 3

Interference with CPSF6 notably suppressed the expression of p-AKT, p-ERK, p-IKKβ and p-p65 in Huh-7 cells, which was partially increased by interference with BTG2 (Fig. 6).

## Discussion

Treatment for HCC has been less than satisfactory, and incidence has increased over the last 20 years due to viral infections such as hepatitis B virus (HBV) and hepatitis C virus (HCV), as well as multiple factors such as alcoholism and aflatoxin [13–15]. Therefore, it is particularly important to further explore the molecular mechanism and new targets in the occurrence and development of HCC.

CPSF6 is localized on human chromosome 12 with a molecular weight of 68 kDa. Previous studies have shown that CPSF6 is associated with a variety of human diseases, including acute myeloid leukemia [16, 17], myeloproliferative neoplasms [18], and human immunodeficiency virus/acquired immunodeficiency syndrome (HIV/AIDS) [19]. Studies have shown that CPSF6 promotes the development of colon cancer [9] and CPSF6 and all core paraspeckles proteins have found to be highly expressed in human breast cancer, which is correlated with poor prognosis [7]. In the present study, CPSF6 expression was confirmed to be upregulated and interference with CPSF6 suppressed the viability and promoted the apoptosis of Huh-7 cells.

BTG1 and BTG2 have been considered as regulators of genotoxic and cellular stress signaling pathways, which can modulate cell apoptosis or survival [20]. In breast carcinoma, low BTG2 expression is related to increased tumor grade, disease progression and poor overall survival. Knockdown of BTG2 causes increased cyclin D1 expression and elevated AKT phosphorylation [21–24]. The increased level of cyclin D1/cyclin E in liver cancer leading to the decreased BTG2 expression causes the increase of tumor grade [25]. In prostate cancer, BTG2 suppression promotes disease progression, therapy resistance, and metastasis [26, 27]. These findings indicate that BTG2 has inhibitory action on tumorigenesis, which is also confirmed in knockout and overexpression of BTG2 in medulloblastoma mice [28, 29]. In this study, BTG2 expression was also decreased in HCC cells. In addition, interference with BTG2 could partially reverse the effects of interference with CPSF6 on the proliferation and apoptosis of HCC cells.

Warburg has found a special phenomenon that in the condition of adequate oxygen supply, tumor cells still show a high level of glycolysis, which produces ATP to supply energy for the metabolic activities of cells, known as aerobic glycolysis or Warburg effect [30, 31]. Tumor cells are more sensitive to glycolysis inhibition [32] and targeting metabolic pathways provides a new strategy for the treatment of tumors [33]. IGF-1R is a transmembrane receptor widely present on the surface of human cells. The expression of IGF-1R is upregulated in HCC, and inhibition of IGF-1R can significantly inhibit the proliferation, migration and invasion of HCC [34]. A study has shown that miR-342-3p directly acts on IGF-1R 3′UTR and reduces the expression of IGF-1R, thereby lowering GLUT1 level, inhibiting glucose uptake of lactic acid to produce ATP, and inducing glycolysis to mitochondrial respiration [35]. miR-199a-5p directly acts on the 3′UTR of HK2, thus inhibiting the generation of glucose consumption lactic acid, reducing cell glucose-6-phosphate (G6P) and ATP levels, affecting cell proliferation and leading to the occurrence of HCC cells [36]. The above findings have confirmed that glycolysis is very important in HCC. Here, we found that interference with CPSF6 inhibited glycolysis of HCC cells, which could partially increase glycolysis by the interference with BTG2.

The tumor suppressor BTG2 served as a regulator of myocardial necrosis by inhibiting AKT/ERK while activating glycogen synthase kinase 3 (GSK3) and cyclophilic protein D [37]. miR-650 binds directly to the 3′UTR of ras-related estrogen-regulated growth inhibitor (RERG) and activates the AKT/ERK/NF-κB pathway mediated by the RERG/PH Domain Leucine-rich Repeat Protein Phosphatase (PHLPP) 2 complex to promote the growth of glioblastoma multiforme [38]. The inhibiting effect of licochalcone A on glycolysis is mainly due to the blockage of AKT signaling pathway, and the inhibition of glycolysis is greatly weakened when AKT is overexpressed [39]. In uncoupling protein 2 (UCP2) knockdown gallbladder cancer cells, proliferation and glycolysis is suppressed and IKKβ and downstream signaling molecules NF-κB/FAK/β-catenin was also downregulated [40]. Of course, interference with CPSF6 blocked the NF-κB/FAK/β-catenin pathway to inhibit the glycolysis of HCC cells by upregulating BTG2 expression.

In conclusion, CPSF6 expression was increased and BTG2 expression was decreased in Huh-7 cells. CPSF6 could bind with BTG2. Interference with CPSF6 promoted apoptosis and inhibited glycolysis of Huh-7 cells by suppressing the NF-κB/FAK/β-catenin pathway, which was partially reversed by BTG2 expression. However, this study is limited for cell experiment. Animal experiment will be used to further confirm the present conclusion in our future study.

## Materials and methods

### Cell culture

Normal hepatocyte (THLE-3) and HCC cells (SK-Hep-1, Huh-7, SNU-449, HCCLM3 and MHCC97H) were obtained from American Type Culture Collection (ATCC). The whole cells were placed in DMEM medium (Thermo Fisher Scientific, Inc.) containing 10% fetal bovine serum (FBS) (Gibco; Thermo Fisher Scientific, Inc.) for routine culture and passage in a biochemical incubator at 37 °C and 5% CO_2_.

### Cell transfection

Synthetic sequences of short hairpin RNA (shRNA) targeting CPSF6 (sh-CPSF6; Shanghai GenePharma co., ltd.), shRNA targeting BTG2 (sh-BTG2) and non-targeting shRNA (shRNA-NC) were inserted into pGPU6/Neo vector (Shanghai GenePharma Co., Ltd.). Trypsin was used to digest the Huh-7 cells to prepare the cell suspension and Huh-7 cells were seeded into a 96-well plate (1 × 10^4^ cells/well). Then, Huh-7 cells were transfected with sh-NC, sh-CPSF6-1/2 and shRNA-BTG2-1/2 in different combinations using Lipofectamine 3000 (Invitrogen; Thermo Fisher Scientific, Inc.). Transfected Huh-7 cells were collected 24 h later for subsequent detection.

### Reverse transcription quantitative real-time polymerase chain reaction (RT-qPCR) analysis

The cells in each group were collected and added with 20 times of the volume of TRIzol reagent (Invitrogen; Thermo Fisher Scientific, Inc.) for lysis. Cells were repeatedly beaten with a disposable syringe to make full lysis. RNA isolation kit (Invitrogen; Thermo Fisher Scientific, Inc.) was used to extract the total RNA, which was reverse transcribed into cDNA, and fluorescence quantitative PCR instrument was used for detection. CPSF6 forward, 5′-GGAGCAGCACCAAATGTTGTC-3′ and reverse, 5′-CTCCCAAAGAATGAACTGCTTC-3′; BTG2 forward, 5′- CATCATCAGCAGGGTGGC-3′ and reverse 5′-CCCAATGCGGTAGGACAC-3′; GAPDH forward, 5′-TGCACCACCAACTGCTTAGC-3′ and reverse 5′-GGCATGGACTGTGGTCATGAG-3′. The 2^−ΔΔCt^ method is used to standardize the relative expression of CPSF6 and BTG2.

### Western blot analysis

The cells were lysed with RIPA lysate (Beyotime) and centrifuged to extract the proteins which were quantified by BCA Protein Assay kit (Beyotime). The proteins were denatured by boiling for 10 min and 60 μg proteins were subjected to 12% SDS-PAGE electrophoresis (Beyotime) per lane. After proteins were transferred to PVDF membranes (EMD Millipore), PVDF membranes were placed in TBST containing 5% skimmed milk powder, which was slowly shaken and sealed for 3 h at room temperature. The primary antibodies [CPSF6 (ab175237; dilution, 1:10000; Abcam), Bcl-2 (ab32124; dilution, 1:1000; Abcam), Bax (ab32503; dilution, 1:1000; Abcam), cleaved-caspase3 (#9664; dilution, 1:1000; Cell signaling technology), cleaved-caspase9 (#20750; dilution, 1:1000; Cell signaling technology), caspase3 (#9662; dilution, 1:1000; Cell signaling technology), caspase9 (#9502; dilution, 1:1000; Cell signaling technology), HK-2 (#2867; dilution, 1:1000; Cell signaling technology), BTG2 (ab197362; dilution, 1:500; Abcam), p-AKT (#4060; dilution, 1:2000; Cell signaling technology), t-AKT (#9272; dilution, 1:1000; Cell signaling technology), p-ERK (ab201015; dilution, 1:1000; Abcam), t-ERK (ab184699; dilution, 1:10000; Abcam), p-IKKβ (#2697; dilution, 1:1000; Cell signaling technology), t-IKKβ (ab97406; dilution, 1:1000; Abcam), p-p65 (ab76302; dilution, 1:1000; Abcam), t-p65 (ab32536; dilution, 1:1000; Abcam) and GAPDH (ab9485; dilution, 1:2500; Abcam)] were added to the membranes for culture at 4 °C overnight and then membranes were washed with TBST for three times. The horseradish peroxidase-linked IgG second antibody (#7074; dilution, 1:1000; Cell Signaling Technology, Inc.) was added to the membranes, which were slowly shaken and cultured for 1 h at room temperature. After washing the membrane, the protein expression was detected by ECL chemiluminescence method, and the expression levels of above proteins were reflected by the ratio of the gray values of above protein bands to that of GAPDH band.

### 3-(4,5-Dimethylthiazol-2-yl)-2,5-diphenyltetrazolium bromide (MTT) assay

Huh-7 cells were digested with 0.25% trypsin (1 mL) for 3 min, inoculated into a 96-well plate with density of 1 × 10^4^ cells/well, and cultured at 37 °C for 24 h. Then, Huh-7 cells were transfected and, respectively, cultured for 24 h, 48 h and 72 h. MTT solution (5 mg/mL) (Beyotime) was added to the plate with 0.02 mL per well. After further culture for 4 h under the original culture conditions, the supernatant in the wells was poured out, and 0.15 mL DMSO was added to each well, which was placed on the oscillator for 10 min to completely dissolve the crystals. The absorbance value of each well with the wavelength of 490 nm was determined by a Multiskan™ Go microplate spectrophotometer (Thermo Fisher Scientifc, Inc.).

### Terminal deoxynucleotidyl transferase (TdT) dUTP nick-end labeling (TUNEL) assay

Huh-7 cells were seeded into a 6-well plate with 1 × 10^5^ cells/well. After cell fusion, cells were transfected accordingly for 24 h, followed by the abandonment of medium. The transfected Huh-7 cells were fixed with 4% formaldehyde for 15 min, dehydrated with 50%, 75%, 95% and 100% ethanol for 5 min, washed with phosphate buffer twice, and treated with 0.5% Triton x-100 for 20 min. TUNEL working solution (Beyotime) was added for incubation at 37 °C for 1 h. Apoptotic cells (green fluorescence staining) were detected by fluorescence microscope (Olympus corporation).

### Detection of glucose and lactate

Huh-7 cells at logarithmic growth stage were inoculated into a 96-well plate with density of 1 × 10^4^ cells/well. After indicated transfection for 24 h, the supernatant of the cells in each group was taken, and the levels of glucose and lactate in the culture supernatant was, respectively, detected with the glucose assay kit (Elabscience) and lactate assay kit (Elabscience).

### RNA binding protein immunoprecipitation (RIP)

Transfected Huh-7 cells were collected and treated according to the RIP kit instructions. Rabbit IgG antibody (#3900; dilution, 1:100;) or anti-CPSF6 antibody (ab175237; dilution, 1:100; Cell Signaling Technology, Inc.) was added to cell lysate and incubated at 4 °C overnight to obtain RNA–protein complex RIP-IgG or RIP-CPSF6. The RNA was extracted and qPCR assay was conducted to detect the enrichment of BTG2 in the RNA–protein complex.

### Statistical analysis

GraphPad Prism 8.0 software (GraphPad Software) was used for statistical analysis of experimental data in the form of mean ± standard deviation (SD) and preparation of figures. One-way analysis of variance (ANOVA) followed by Tukey’s post hoc test and unpaired *t*-test, respectively, analyzed the differences in the multiple groups and two groups. Statistical significance was considered to be *P* < 0.05.

## Supplementary Information

**Additional file 1: Figure S1.** CPSF6 in liver hepatocellular carcinoma (LIHC). (A) CPSF6 with 374 cancer and 50 normal samples in LIHC. (B) Overall survival for CPSF6 in LIHC.

**Additional file 2: Figure S2.** Overall survival for BTG2 in LIHC.

## Data Availability

The experimental data will be available on the request.

## References

[1] Han K, Kim JH, Ko GY, Gwon DI, Sung KB (2016). Treatment of hepatocellular carcinoma with portal venous tumor thrombosis: a comprehensive review. World J Gastroenterol.

[2] Baghy K, Tátrai P, Regős E, Kovalszky I (2016). Proteoglycans in liver cancer. World J Gastroenterol.

[3] Villanueva A (2019). Hepatocellular carcinoma. N Engl J Med.

[4] Hartke J, Johnson M, Ghabril M (2017). The diagnosis and treatment of hepatocellular carcinoma. Semin Diagn Pathol.

[5] Clark T, Maximin S, Meier J, Pokharel S, Bhargava P (2015). Hepatocellular carcinoma: review of epidemiology, screening, imaging diagnosis, response assessment, and treatment. Curr Probl Diagn Radiol.

[6] Hardy JG, Norbury CJ (2016). Cleavage factor Im (CFIm) as a regulator of alternative polyadenylation. Biochem Soc Trans.

[7] Binothman N, Hachim IY, Lebrun JJ, Ali S (2017). CPSF6 is a clinically relevant breast cancer vulnerability target: role of CPSF6 in breast cancer. EBioMedicine.

[8] Wang BJ, Liu DC, Guo QY, Han XW, Bi XM, Wang H, Wu ZS, Wu WY (2020). NUDT21 suppresses breast cancer tumorigenesis through regulating CPSF6 expression. Cancer Manag Res.

[9] Larriba MJ, Casado-Vela J, Pendás-Franco N, Peña R, de Herreros AG, Berciano MT, Lafarga M, Casal JI, Muñoz A (2010). Novel snail1 target proteins in human colon cancer identified by proteomic analysis. PLoS ONE.

[10] Xie Y, Du J, Liu Z, Zhang D, Yao X, Yang Y (2019). MiR-6875-3p promotes the proliferation, invasion and metastasis of hepatocellular carcinoma via BTG2/FAK/Akt pathway. J Exp Clin Cancer Res.

[11] Zhang SJ, Ma J, Wu JC, Hao ZZ, Zhang YA, Zhang YJ (2020). Circular RNA circCRIM1 suppresses lung adenocarcinoma cell migration, invasion, EMT, and glycolysis through regulating miR-125b-5p/BTG2 axis. Eur Rev Med Pharmacol Sci.

[12] Mao B, Xiao H, Zhang Z, Wang D, Wang G (2015). MicroRNA-21 regulates the expression of BTG2 in HepG2 liver cancer cells. Mol Med Rep.

[13] Wang Y, Cao J, Zhang S, Sun L, Nan Y, Yao H, Fan J, Zhu LY, Yu L (2019). MicroRNA-802 induces hepatitis B virus replication and replication through regulating SMARCE1 expression in hepatocellular carcinoma. Cell Death Dis.

[14] Wong CC, Tse AP, Huang YP, Zhu YT, Chiu DK, Lai RK, Au SL, Kai AK, Lee JM, Wei LL (2014). Lysyl oxidase-like 2 is critical to tumor microenvironment and metastatic niche formation in hepatocellular carcinoma. Hepatology.

[15] Vaira V, Roncalli M, Carnaghi C, Faversani A, Maggioni M, Augello C, Rimassa L, Pressiani T, Spagnuolo G, Di Tommaso L (2015). MicroRNA-425-3p predicts response to sorafenib therapy in patients with hepatocellular carcinoma. Liver Int.

[16] Liu T, Wen L, Yuan H, Wang Y, Yao L, Xu Y, Cen J, Ruan C, Wu D, Chen S (2018). Identification of novel recurrent CPSF6-RARG fusions in acute myeloid leukemia resembling acute promyelocytic leukemia. Blood.

[17] Qin YZ, Huang XJ, Zhu HH (2018). Identification of a novel CPSF6-RARG fusion transcript in acute myeloid leukemia resembling acute promyelocytic leukemia. Leukemia.

[18] Naumann N, Schwaab J, Metzgeroth G, Jawhar M, Haferlach C, Göhring G, Schlegelberger B, Dietz CT, Schnittger S, Lotfi S (2015). Fusion of PDGFRB to MPRIP, CPSF6, and GOLGB1 in three patients with eosinophilia-associated myeloproliferative neoplasms. Genes Chromosomes Cancer.

[19] Henning MS, Dubose BN, Burse MJ, Aiken C, Yamashita M (2014). In vivo functions of CPSF6 for HIV-1 as revealed by HIV-1 capsid evolution in HLA-B27-positive subjects. PLoS Pathog.

[20] Yuniati L, Scheijen B, van der Meer LT, van Leeuwen FN (2019). Tumor suppressors BTG1 and BTG2: Beyond growth control. J Cell Physiol.

[21] Kawakubo H, Brachtel E, Hayashida T, Yeo G, Kish J, Muzikansky A, Walden PD, Maheswaran S (2006). Loss of B-cell translocation gene-2 in estrogen receptor-positive breast carcinoma is associated with tumor grade and overexpression of cyclin d1 protein. Cancer Res.

[22] Kawakubo H, Carey JL, Brachtel E, Gupta V, Green JE, Walden PD, Maheswaran S (2004). Expression of the NF-kappaB-responsive gene BTG2 is aberrantly regulated in breast cancer. Oncogene.

[23] Takahashi F, Chiba N, Tajima K, Hayashida T, Shimada T, Takahashi M, Moriyama H, Brachtel E, Edelman EJ, Ramaswamy S, Maheswaran S (2011). Breast tumor progression induced by loss of BTG2 expression is inhibited by targeted therapy with the ErbB/HER inhibitor lapatinib. Oncogene.

[24] van de Vijver MJ, He YD, van’t Veer LJ, Dai H, Hart AA, Voskuil DW, Schreiber GJ, Peterse JL, Roberts C, Marton MJ (2002). A gene-expression signature as a predictor of survival in breast cancer. N Engl J Med.

[25] Zhang Z, Chen C, Wang G, Yang Z, San J, Zheng J, Li Q, Luo X, Hu Q, Li Z, Wang D (2011). Aberrant expression of the p53-inducible antiproliferative gene BTG2 in hepatocellular carcinoma is associated with overexpression of the cell cycle-related proteins. Cell Biochem Biophys.

[26] Coppola V, Musumeci M, Patrizii M, Cannistraci A, Addario A, Maugeri-Saccà M, Biffoni M, Francescangeli F, Cordenonsi M, Piccolo S (2013). BTG2 loss and miR-21 upregulation contribute to prostate cell transformation by inducing luminal markers expression and epithelial-mesenchymal transition. Oncogene.

[27] Jalava SE, Urbanucci A, Latonen L, Waltering KK, Sahu B, Jänne OA, Seppälä J, Lähdesmäki H, Tammela TL, Visakorpi T (2012). Androgen-regulated miR-32 targets BTG2 and is overexpressed in castration-resistant prostate cancer. Oncogene.

[28] Farioli-Vecchioli S, Tanori M, Micheli L, Mancuso M, Leonardi L, Saran A, Ciotti MT, Ferretti E, Gulino A, Pazzaglia S, Tirone F (2007). Inhibition of medulloblastoma tumorigenesis by the antiproliferative and pro-differentiative gene PC3. Faseb J.

[29] Farioli-Vecchioli S, Cinà I, Ceccarelli M, Micheli L, Leonardi L, Ciotti MT, De Bardi M, Di Rocco C, Pallini R, Cavallaro S, Tirone F (2012). Tis21 knock-out enhances the frequency of medulloblastoma in Patched1 heterozygous mice by inhibiting the Cxcl3-dependent migration of cerebellar neurons. J Neurosci.

[30] Vander Heiden MG, Cantley LC, Thompson CB (2009). Understanding the Warburg effect: the metabolic requirements of cell proliferation. Science.

[31] Hanahan D, Weinberg Robert A (2011). Hallmarks of cancer: the next generation. Cell.

[32] Warburg O (1956). On the origin of cancer cells. Science.

[33] Luengo A, Gui DY, Vander Heiden MG (2017). Targeting metabolism for cancer therapy. Cell Chem Biol.

[34] Bie CQ, Liu XY, Cao MR, Huang QY, Tang HJ, Wang M, Cao GL, Yi TZ, Wu SL, Xu WJ, Tang SH (2016). Lentivirus-mediated RNAi knockdown of insulin-like growth factor-1 receptor inhibits the growth and invasion of hepatocellular carcinoma via down-regulating midkine expression. Oncotarget.

[35] Liu W, Kang L, Han J, Wang Y, Shen C, Yan Z, Tai Y, Zhao C (2018). miR-342-3p suppresses hepatocellular carcinoma proliferation through inhibition of IGF-1R-mediated Warburg effect. Onco Targets Ther.

[36] Guo W, Qiu Z, Wang Z, Wang Q, Tan N, Chen T, Chen Z, Huang S, Gu J, Li J (2015). MiR-199a-5p is negatively associated with malignancies and regulates glycolysis and lactate production by targeting hexokinase 2 in liver cancer. Hepatology.

[37] Choi YW, Park TJ, Kim HS, Lim IK (2013). Signals regulating necrosis of cardiomyoblast by BTG2(/TIS21/PC3) via activation of GSK3β and opening of mitochondrial permeability transition pore in response to H2O2. Biochem Biophys Res Commun.

[38] Jin S, Li X, Dai Y, Li C, Wang D (2020). NF-κB-mediated miR-650 plays oncogenic roles and activates AKT/ERK/NF-κB pathways by targeting RERG in glioma cells. Cellular Oncol.

[39] Wu J, Zhang X, Wang Y, Sun Q, Chen M, Liu S, Zou X (2018). Licochalcone A suppresses hexokinase 2-mediated tumor glycolysis in gastric cancer via downregulation of the Akt signaling pathway. Oncol Rep.

[40] Yu J, Shi L, Lin W, Lu B, Zhao Y (2020). UCP2 promotes proliferation and chemoresistance through regulating the NF-κB/β-catenin axis and mitochondrial ROS in gallbladder cancer. Biochem Pharmacol.

