# Supplementary figures and images for: Ribonucleic acid-binding protein CPSF6 promotes glycolysis and suppresses apoptosis in hepatocellular carcinoma cells by inhibiting the BTG2 expression
Source: Biomed Eng Online. 2021 Jul 3;20:67. doi: 10.1186/s12938-021-00903-6 (PMC8254334; doi:10.1186/s12938-021-00903-6)

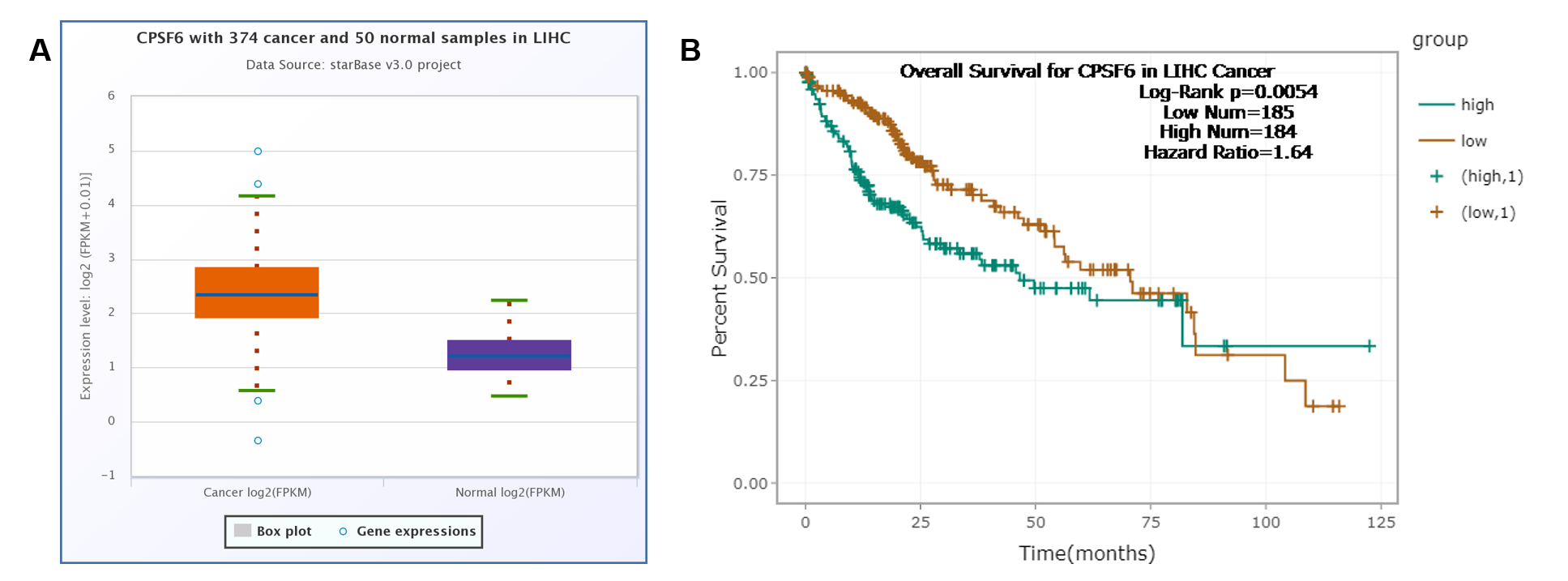

Supplement: Supplementary file 1 — Additional file 1: Figure S1. CPSF6 in liver hepatocellular carcinoma (LIHC). (A) CPSF6 with 374 cancer and 50 normal samples in LIHC. (B) Overall survival for CPSF6 in LIHC. [file 12938_2021_903_MOESM1_ESM.tif]

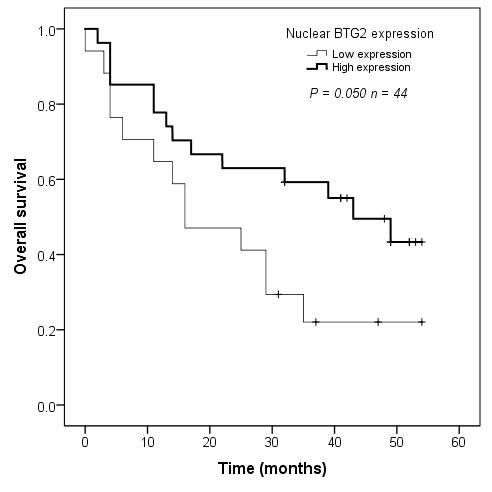

Supplement: Supplementary file 2 — Additional file 2: Figure S2. Overall survival for BTG2 in LIHC. [file 12938_2021_903_MOESM2_ESM.tif]
